# Jaagsiekte sheep retrovirus infection of lung slice cultures

**DOI:** 10.1186/s12977-015-0157-5

**Published:** 2015-04-09

**Authors:** Chris Cousens, Charline Alleaume, Esther Bijsmans, Henny M Martineau, Jeanie Finlayson, Mark P Dagleish, David J Griffiths

**Affiliations:** Moredun Research Institute, Pentlands Science Park, Bush Loan, Penicuik Edinburgh, UK; Department of Pathology and Infectious Diseases, Royal Veterinary College, Hawkshead Lane, North Mymms, Hatfield, Hertfordshire, UK

**Keywords:** Jaagsiekte, Ovine pulmonary adenocarcinoma, Precision-cut lung slice, Retrovirus, Tumor model

## Abstract

**Background:**

Jaagsiekte sheep retrovirus (JSRV) is the causative agent of ovine pulmonary adenocarcinoma (OPA), a transmissible neoplastic disease of sheep. OPA is an economically important veterinary disease and is also a valuable naturally occurring animal model of human lung cancer, with which it shares a similar histological appearance and the activation of common cell signaling pathways. Interestingly, the JSRV Env protein is directly oncogenic and capable of driving cellular transformation *in vivo* and *in vitro*. Previous studies of JSRV infection in cell culture have been hindered by the lack of a permissive cell line for the virus. Here, we investigated the ability of JSRV to infect slices of ovine lung tissue cultured *ex vivo*.

**Results:**

We describe the use of precision cut lung slices from healthy sheep to study JSRV infection and transformation *ex vivo*. Following optimization of the culture system we characterized JSRV infection of lung slices and compared the phenotype of infected cells to natural field cases and to experimentally-induced OPA tumors from sheep. JSRV was able to infect cells within lung slices, to produce new infectious virions and induce cell proliferation. Immunohistochemical labeling revealed that infected lung slice cells express markers of type II pneumocytes and phosphorylated Akt and ERK1/2. These features closely resemble the phenotype of natural and experimentally-derived OPA in sheep, indicating that lung slice culture provides an authentic *ex vivo* model of OPA.

**Conclusions:**

We conclude that we have established an *ex vivo* model of JSRV infection. This model will be valuable for future studies of JSRV replication and early events in oncogenesis and provides a novel platform for studies of JSRV-induced lung cancer.

## Background

Lung cancer is the leading cause of cancer deaths worldwide, causing over 1.6 million deaths in 2012 [1]. Patients with lung cancer typically do not present with symptoms until the tumor is well established and has already metastasized, which contributes to the low survival rate [2]. Greater understanding of the molecular and cellular basis of lung cancer is required to drive improvements in prevention, diagnosis, prognosis and treatment. Animal models of lung cancer, especially in mice have provided a wealth of information on the molecular basis of cancer (reviewed by [3]). However, murine models do not fully mimic the physiology of the human lung and alternative animal species may offer novel insights into pulmonary biology. For example, the sheep has been suggested to offer advantages over murine models due to the closer anatomical similarity to human lungs with respect to size and structure [4].

Ovine pulmonary adenocarcinoma (OPA; also known as jaagsiekte) is a common lung cancer of sheep caused by Jaagsiekte sheep retrovirus (JSRV) (reviewed by [5-7]). OPA is an economically important disease of sheep in many countries and has also been proposed as a model of human lung cancer; in particular for relatively non-invasive forms such as lepidic-predominant adenocarcinoma (formerly bronchioloalveolar carcinoma) [8]. Although OPA has a known viral etiology [9], no infectious cause has been found for human lung cancer [10]. Nevertheless, OPA represents an important model system that provides opportunities to study early events in lung cancer tumorigenesis and to develop methods for early diagnosis. Notably, experimental reproduction of OPA can be achieved by infection of young lambs with JSRV harvested from OPA-affected animals or by virus produced *in vitro* using an infectious molecular clone [9,11,12]. This experimental system provides an excellent disease model for studying OPA pathogenesis.

The mechanism of oncogenesis by JSRV has been the subject of a number of studies (reviewed by [13]), which have demonstrated that the envelope (Env) protein of JSRV is oncogenic and expression of Env alone is sufficient to transform cell lines *in vitro* [14,15] and to induce tumors in immunosuppressed mice or in lambs [16,17]. In studies aimed at examining early events in JSRV infection and transformation, JSRV-infected cells have been characterized in experimentally infected lambs 10 days after infection [18,19]. However, performing such studies *in vivo* is very laborious due to the difficulty of finding the small number of JSRV-infected cells in the ovine lung so soon after infection [18].

*In vivo* reproduction of OPA, of course, has cost and ethical implications and where possible replacement with appropriate *in vitro* systems is desirable. However, analysis of JSRV infection and transformation *in vitro* has been hindered by the lack of a permissive cell line that can support efficient JSRV replication. *In vivo*, JSRV is known to infect a number of diverse cell types, including epithelial cells in the lung and myeloid and lymphoid cells in the lymphoreticular system [20,21]. However, if replication of the virus in lymphocytes and monocyte/macrophages occurs, it is at a very low level as JSRV protein expression in these cells has been detected only rarely. In the sheep lung, JSRV is known to predominantly infect proliferating type II pneumocytes [19] and, to a lesser extent, club cells [18,22] (formerly known as Clara cells [23]) but these cell types do not maintain their differentiated state in culture unless grown in 3D culture systems such as matrigel matrix and transwell dishes [24-26], which reflects the importance of polarization for maintaining epithelial cell phenotype. A system that can support JSRV replication *in vitro* would greatly benefit studies on OPA pathogenesis.

Here, we describe the use of precision-cut lung slices from healthy sheep to study JSRV infection and transformation *ex vivo*. This system allows the maintenance of type II pneumocytes and other pulmonary cell types in culture and, because the tissue architecture and diversity of cell types are maintained, provides a more authentic representation of the *in vivo* lung than cell lines grown as monolayers or in 3D matrices [27,28]. Following optimization of the culture system we demonstrated that JSRV replicates in ovine lung slices and that the phenotype of infected cells reproduces those observed in natural field cases of OPA (OPA-N) and *in vivo* experimentally-induced OPA (OPA-E) tumors. These data confirm lung slice culture as an authentic *in vitro* system for studying early events in JSRV infection and pulmonary cell transformation.

## Results

### Establishment of an ovine lung slice culture system

Precision-cut lung slices were prepared from normal healthy ovine lungs using a procedure similar to those that have been used successfully in other species [27]. Although lung slice cultures provide a closer *in vitro* model of the lung than monolayer cultures [28], *ex vivo* culture is nevertheless likely to have significant effects on the tissue and therefore the first question addressed was how the lung slices change over time in culture. The viability of the lung slices, as judged by visible ciliary activity was maintained for at least 21 days. Cell viability was also assessed by staining the cytoplasm of live cells with a green fluorescent dye, and the nuclei of membrane-compromised cells with a red fluorescent dye. This confirmed that during the first week in culture most cells in the lung slices were alive although dead cells were evident around the peripheral cut surfaces (data not shown). Subsequently, the number of dead cells increased but the background yellow/green autofluorescence of the lung slices also increased so it became difficult to visualize the live/dead staining after 2–3 weeks in culture despite visible ciliary activity.

**Figure 1:**
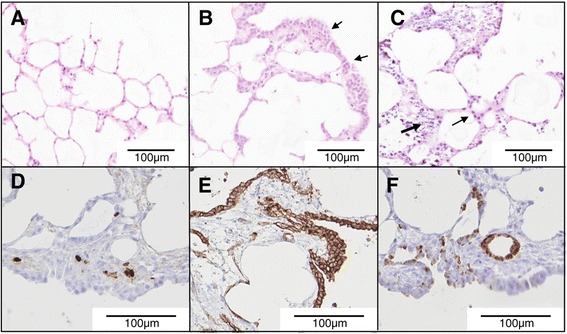
**Viability and structure of cultured ovine lung slices.** Hematoxylin and eosin (HE) stained uninfected lung slices at 0 **(A)**, 16 **(B)** and 42 **(C)** dpi. In panel B the arrows indicate areas of “thickening” around the edges of the lung slice. In panel C the small arrow indicates epithelial cells and the large arrow indicates necrotic interstitial cells. **D, E, F)** IHC of d16 lung slices labeled with anti-Ki-67 **(D)**, anti-pan-cytokeratin **(E)**, and anti-DC-LAMP **(F)**. Lung slices **D**, **E** and **F** were treated with the replication-defective JSRV-∆RT virus.

Morphological changes due to hyper-cellularity were noticeable from around day 8 in culture, and were particularly marked around the cut edges of the slices (Figure 1A, B). Similar gross changes were seen both in JSRV-infected or uninfected lung slices demonstrating that the aberrant growth was not due to JSRV infection but instead appears to be a reaction of the tissue to processing and/or *in vitro* culture. After an extended time in culture (42 days) the epithelial cells continued to appear histologically normal whereas interstitial cells appeared degenerate (Figure 1C). IHC labeling with an antibody to the proliferation marker Ki-67 suggested that the structural changes in the first weeks of culture were due to proliferation of cells in the interstitial compartment (Figure 1D). There was also an increase in cuboidal cells lining the alveoli which were positive for pan-cytokeratin (an epithelial cell marker) (Figure 1E) and DC-LAMP (type II pneumocytes) (Figure 1F), indicative of type II pneumocyte hyperplasia, which may be a response of the tissue to injury caused by slicing. This analysis indicated that ovine lung slices can survive in culture for at least 6 weeks whilst retaining many of the characteristics of normal lung tissue.

### JSRV infection of lung slices

We next determined whether JSRV can infect and replicate in cultured ovine lung slices. Lung slices were prepared and JSRV was added to the culture medium. At 4, 8, 12 and 16 days post infection (dpi) the supernatant (culture medium) was harvested and analyzed for the presence of JSRV RNA by RT-qPCR. Negative controls included uninfected lung slices and lung slices infected with a replication-defective JSRV mutant that carries an inactivating mutation in the active site of reverse transcriptase (JSRV-ΔRT). Slices of lung tumor prepared from OPA-N lung were used as positive controls.

**Figure 2:**
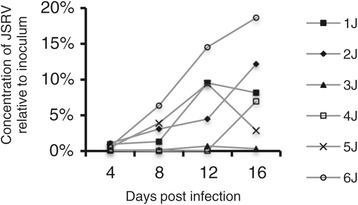
**Detection of JSRV RNA in lung slice supernatants indicates replication of JSRV.** JSRV was added to lung slices and medium changed daily. The results show JSRV RNA content of 24 h supernatants (pooled from 4 wells) presented as a percentage of the inoculum (100%) for lung slices prepared from 6 animals of a range of ages; 9 months old (1 J and 2 J), 10 years old (3 J), 2 years old (4 J), and 5 days old (5 J and 6 J). Results for the equivalent JSRV-∆RT-treated lung slices are not shown as these were all negative after day 4. Control lung slices to which no JSRV was added were negative throughout. The results shown here are for the lung slices from the same experiments that provided the lung slices for serial sections.

Culture supernatants from wells containing tissue slices of OPA-N were always strongly positive (Ct 20–24) by RT-qPCR for JSRV RNA, while supernatants from wells containing uninfected lung slices were always negative. RNA from replication-defective JSRV-∆RT was detectable at low levels (0.01-0.1% of inoculum) in lung slice supernatant up to 4 dpi. This most likely results from residual input virus that has non-specifically bound to the lung slices or the culture plate. Thereafter, the supernatants of JSRV-∆RT-treated lung slices were consistently negative by JSRV RT-qPCR, indicating that residual input virus was not present in samples taken at 8 dpi and later. In contrast, JSRV RNA was readily detected in the supernatant of JSRV-infected lung slices from 4 or 8 dpi and the concentration increased further towards 12 and 16 dpi (Figure 2). However, there was large variability in the quantity of virus detected in the supernatant between experiments (*i.e.*, using lung slices sourced from different animals) despite the use of a standardized infection and analysis protocol.

**Figure 3:**
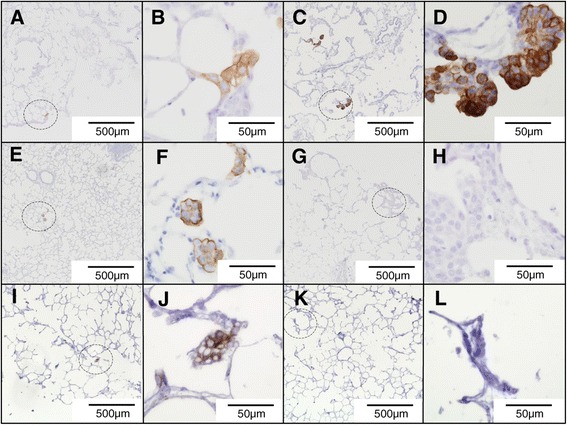
**JSRV-infected lung slice and early experimental OPA show a similar pattern of JSRV-SU labeling.** Sections of JSRV-infected tissue were labeled with a monoclonal antibody to JSRV Env (SU) by IHC. Positive labeling is shown by brown pigment. **A, B)** JSRV-infected lung slice 8 dpi; **C, D)** JSRV-infected lung slice 16 dpi; **E, F)** Lung tissue from lambs experimentally infected with JSRV 10 dpi; **G, H)** Lung slices infected with control virus JSRV-∆RT 16 dpi showed no labeling. **I, J)** Lung slices infected with pooled supernatant of previous JSRV-infected lung slices 12 dpi. **K, L)** Lung slices infected with pooled supernatant of previous JSRV-∆RT infected lung slices 12 dpi. Dashed circles in **A**, **C**, **E**, **G**, **I** and **K** indicate the regions shown at higher magnification in **B**, **D**, **F**, **H**, **J** and **L**, respectively.

We then examined JSRV-infected lung slices by IHC using an antibody to the JSRV Env (SU) protein [29]. Foci of SU-positive cells were detectable from 4 dpi onwards (Figure 3A-F), but the number of positive cells per section was highly variable even within a single experiment (*i.e.*, replicate lung slices from a single donor animal) and the proportion of positive sections varied between experiments (*i.e.*, lung slices from different donor animals). For example, in the experiment shown in Figure 3A-D, at 8 dpi only 1 group of SU-positive cells was detected from sections of 4 replicate lung slices whereas at 12 or 16 dpi two or three groups of cells expressing JSRV SU were visible in the sections of 3 of the 4 lung slice replicates. The histological appearance of clusters of labeled cells in JSRV-infected lung slices (Figure 3A-D) was indistinguishable from early nodules in OPA-E (Figure 3E-F). SU-labeling of cells was localized in the cytoplasm and at the cell surface with stronger labeling at the apical pole. SU-labeling was not seen in any of the uninfected lung slices nor in JSRV-∆RT-treated lung slices (Figure 3G, H), thus confirming the specificity of the anti-SU antibody in IHC.

The RT-qPCR and IHC data indicate that JSRV can infect cells in ovine lung slices and express viral proteins and release viral particles. We next asked whether the JSRV released by infected lung slices is also infectious. Supernatants from JSRV-infected and JSRV-∆RT-treated lung slices were harvested and used to infect new lung slice cultures. The supernatants from JSRV-infected lung slices, but not JSRV-∆RT-treated lung slices, gave rise to JSRV-positive cells as shown by IHC for SU (Figure 3I-L). These results confirm that JSRV produced by lung slice cultures is infectious. Together, these data indicate that JSRV is able to infect and replicate in ovine lung slices cultured *in vitro* thereby providing a system to examine early events following infection.

### JSRV infects type 2 pneumocytes in ovine lung slices

**Figure 4:**
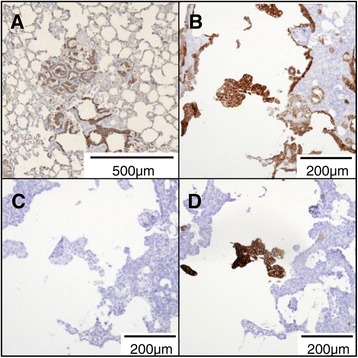
**IHC with anti-pancytokeratin antibody labels respiratory epithelial cells and this co-localises with JSRV-positive tumor cells in the lungs of OPA-positive sheep and JSRV-positive lung slice cells. A)** OPA-N; **B**, **C**, **D)** Serial adjacent sections of JSRV-infected lung slice 16 dpi labeled with **A, B)** anti-pancytokeratin (respiratory epithelium); **C)** rabbit IgG (IHC negative control); **D)** anti-SU (JSRV-positive cells).

**Figure 5:**
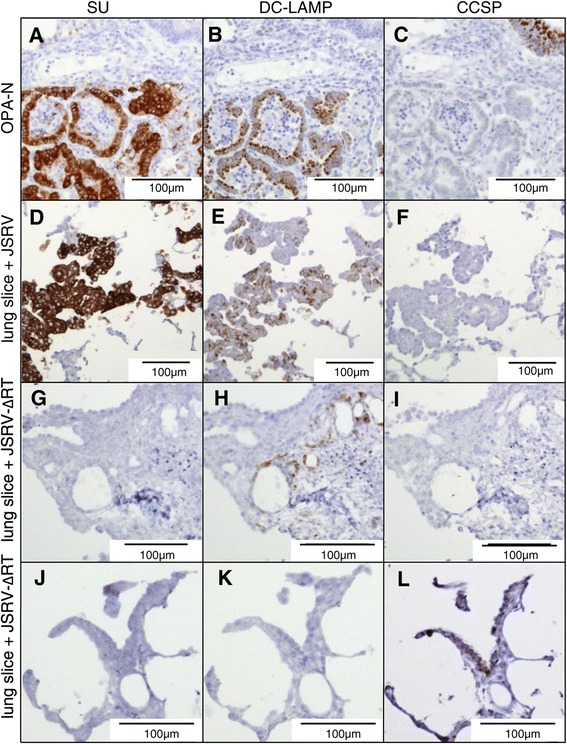
**SU-positive cells in JSRV-infected lung slices are CCSP negative and DC-LAMP positive.** Serial sections of **A-C)** OPA-N; **D-F)** JSRV-infected lung slice,16 dpi and **G-L)** JSRV-∆RT-infected lung slice 16 dpi were subjected to IHC for JSRV SU and for respiratory epithelial cell markers: **A**, **D**, **G**, **J)** anti-JSRV SU; **B**, **E**, **H**, **K)** anti-DC-LAMP; **C**, **F**, **I**, **L)** anti-CCSP; Figure 5A and G/J show positive and negative controls, respectively, for JSRV SU-labeling. Note areas of co-labeling with markers for OPA tumor cells and type II pneumocytes **(A/B** and **D/E)** but no co-labeling with the marker for club cells **(C** and **F)**.

The identity of cell types infected and transformed by JSRV in OPA has attracted interest as these may also represent the cells of origin of other lung cancers. We therefore examined the phenotype of JSRV-infected cells within cultured lung slices using IHC on serial adjacent sections labeled with the anti-JSRV SU antibody and antibodies to respiratory epithelial cellular markers (Table 1, Figures 4 and 5). A panel of six lung slices that contained many SU-positive foci was selected for analysis. Sections of lung from OPA-N were analyzed in parallel for comparison.

**Table 1:** **Summary of antibodies used for IHC**

**Antibody target antigen**	**Cell target**	**Dilution**	**Antibody type**	**Source**	**Positive control tissue**
Cytokeratin	Pan-respiratory epithelia	1/5000	Mouse mab*	DAKO M3515	normal sheep lung
JSRV Env (SU)	JSRV-infected	1/800	2 Mouse mabs	[16]	OPA positive sheep lung
Club cell specific protein (CCSP)	Club cell	1/20,000	Rabbit polyclonal	[18,19]	normal sheep lung
Dendritic cell lysosome-associated membrane glycoprotein (DC-LAMP)	Type II pneumocytes	1/1,000	Rat mab	Dendritics Clone 1010E1.01	normal sheep lung
Ki-67	Dividing cells	1/1,000	Mouse mab	DAKO M7240	normal sheep lung
Phospho-ERK1/2 (Thr202/Tyr204)	Activated ERK1/2	1/600	Rabbit mab	Cell Signaling NEB(UK) 4370S	normal sheep placenta [62]
Phospho-Akt (Ser473) (D9E)	Activated Akt	1/400	Rabbit mab	Cell Signaling NEB(UK) 4060S	normal sheep placenta [62]
Normal rabbit serum	Negative control	matched to primary	Rabbit polyclonal	In house	NA
Normal mouse IgG	Negative control	matched to primary	Mouse	Sigma I5381	NA
Normal rabbit IgG	Negative control	matched to primary	Rabbit	Sigma I5006	NA

*mab: monoclonal antibody NA: not applicable.

In the lung, cytokeratins are a marker for pulmonary conducting and respiratory airway epithelial cells and anti-pancytokeratin antibody labels the cells lining the trachea, bronchi and bronchioles and pneumocytes. Using IHC with an anti-pancytokeratin antibody, we found that this also labeled tumor cells in OPA-N (Figure 4A). In serial sections of JSRV-infected lung slices, cells labeling with anti-pancytokeratin (Figure 4B) co-aligned with SU-positive cells (Figure 4D) confirming that the JSRV-infected cells in lung slices were of epithelial origin.

To determine whether JSRV infects type II pneumocytes in lung slices, we initially used an antibody to surfactant protein C (SP-C), which is a specific marker of those cells. Although this antibody is highly specific on ovine lung tissue [18,30], it produced non-specific labeling on lung slices (not shown). As an alternative, we used an antibody to DC-LAMP (CD208), which has been shown previously to be a marker of type II pneumocytes [31,32]. On OPA-N lung tissue, DC-LAMP labeling was observed in the cytoplasm of type II pneumocytes and in OPA tumor cells (Figure 5B). On the lung slices, anti-DC-LAMP labeled cells in the expected anatomical locations and with a morphology typical of type II pneumocytes (Figure 5H). In addition, for JSRV-infected lung slices all 6 sets of serial sections analyzed exhibited co-alignment of cells labeling with anti-SU and cells with positive labeling for DC-LAMP (Figure 5D, E). The DC-LAMP labeling observed was granular and cytoplasmic varying from weak to intense.

In normal and OPA-N lung, an antibody recognizing club cell-specific protein (CCSP) labeled the cytoplasm of cells in the bronchiolar epithelium consistent with the expected location and morphology of club cells (*i.e.*, non-ciliated cells positioned between the ciliated cells) (Figure 5C). CCSP-positive cells were not observed in OPA-N tumor nodules (Figure 5C). In the lung slices, anti-CCSP labeled club cells in the bronchiolar epithelium (Figure 5L) but did not label SU-positive cells in any of the JSRV-infected lung slices (Figure 5F). Taken together, the IHC results indicate that JSRV-infected cells in lung slices are of epithelial origin and express markers of type II pneumocytes, which is the same as the phenotype of JSRV-infected cells in OPA-N and OPA-E.

### Proliferation and transformation of JSRV-infected cells

**Figure 6:**
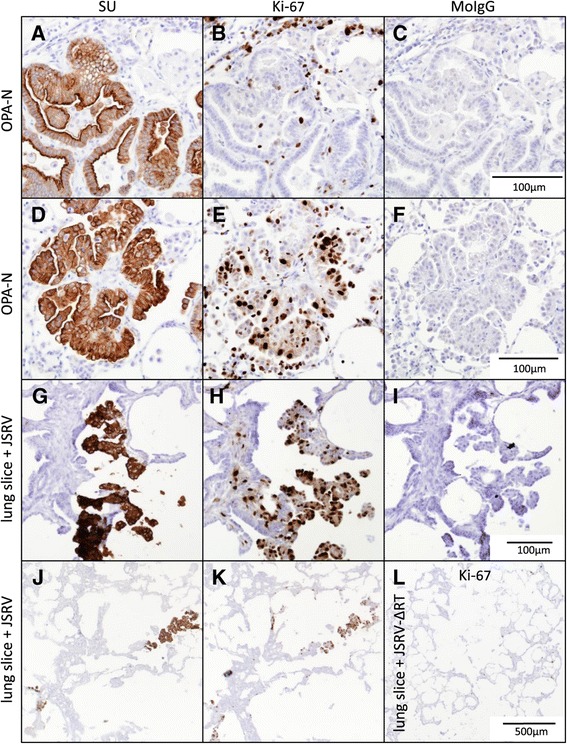
**Cell proliferation is increased in SU-positive cells in JSRV-infected lung slices and in some, but not all, OPA tumor nodules.** Serial sections were labeled with anti-JSRV SU (OPA tumor cells), anti-Ki-67 (proliferation marker), and with murine IgG (MoIgG) (negative control) antibodies. **A-F)** OPA-N; **G-K)** JSRV-infected lung slice (16 dpi); **L)** JSRV-∆RT-infected lung slice (16 dpi). Each scale bar is representative for the whole row.

Abnormally increased cell division is a fundamental trait of cell transformation and tumor development [33,34]. Therefore, we investigated whether increased cell division was associated with JSRV infection using an antibody to the proliferation marker Ki-67 (Figure 6). On sections of OPA-N tissue, anti-Ki-67 was found to label the nuclei of neutrophils and a moderate number of endothelial cells and fibroblasts (not shown). In general, only a small proportion (<10%) of OPA tumor cells were labeled but in some tumor nodules a higher proportion (>60%) of labeled cells was seen. For example, in the OPA nodule shown in Figure 6A-C approximately 5% of tumor cells labeled with anti-Ki-67 whereas a different OPA nodule from the same animal, shown in Figure 6D-F, showed more than 50% of tumor cells labeled positively for Ki-67. In JSRV-infected lung slices, the majority of SU-positive cells were also Ki-67 positive (Figure 6G, H) showing that a large number of the JSRV-infected cells were proliferating. Indeed, at low magnification SU-positive groups of cells could easily be aligned with Ki-67 positive groups of cells (Figure 6J, K) whereas JSRV-ΔRT-infected lung slices did not show similar areas of dense Ki-67 labeling (Figure 6L).

Studies in cell lines have identified that JSRV Env activates a number of cellular signaling pathways involved in neoplastic transformation, including the Akt-mTOR and Ras-Raf-MEK-ERK pathways (reviewed by [13]). Phosphorylation of Akt or ERK1/2, respectively, are indicative of activation of these signaling pathways. In order to examine their activation in JSRV-infected lung slices, IHC with antibodies against phospho-Akt (P-Akt) and phospho-ERK1/2 (P-ERK1/2) was employed after first optimizing on ovine placental tissue (Table 1).

**Figure 7:**
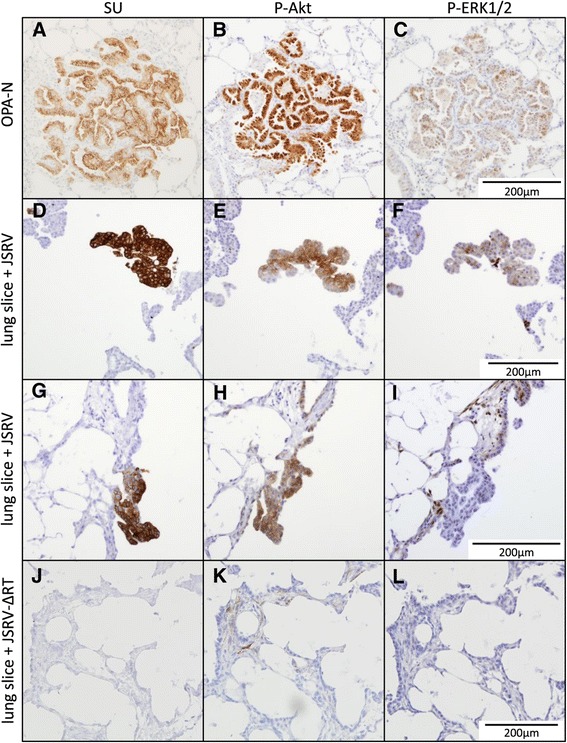
**JSRV-infected lung slice cells are positive for P-Akt and either positive or negative for P-ERK1/2 by IHC.** Serial sections were labeled with antibodies against JSRV SU, P-Akt and P-ERK1/2. **A-C)** OPA-N; **D-F)** JSRV-infected lung slice (16 dpi); **G-I)** JSRV-infected lung slice (20 dpi); **J-L)** JSRV-∆RT-treated lung slice (16 dpi). Note consistent co-labeling of P-Akt and SU positive cells compared to inconsistent labeling of P-ERK1/2 for OPA-N and JSRV-infected lung slices and lack of labeling in JSRV-∆RT-treated lung slice. Each scale bar is representative for the whole row.

IHC on OPA-N indicated that 50-100% of OPA tumor cells labeled positively with anti-P-Akt (nuclear and cytoplasmic labeling) (Figure 7B), with variation between different tumor nodules both in the proportion of positive cells and the intensity of labeling. There was faint background labeling of bronchiolar cells and macrophages in some sections. The variable intensity of labeling between different tumor nodules remained consistent even when applying different concentrations of primary antibody, indicating that the variation was not due to limiting amounts of primary antibody but most likely reflects different concentrations of P-Akt present within different tumor cells. On JSRV-infected lung slices, IHC of serial sections showed that SU-positive cells co-aligned with strong labeling for P-Akt (Figure 7D, E, G, and H). In contrast, lung slices infected with JSRV-ΔRT showed P-Akt labeling only in occasional interstitial cells (Figure 7K).

Labeling of OPA-N lung sections for P-ERK1/2 identified positive tumor cells in 11 of 12 cases analyzed (Figure 7C) and was localized either to the nucleus alone or to the nucleus and cytoplasm. The proportion of tumor cells labeling positively for P-ERK1/2 varied between individual nodules in the same section from none to a low proportion (<10%) in most nodules, through to 100% of tumor cells in some nodules. The intensity of labeling ranged from weak to very strong. In addition, some labeling was observed in endothelial cells, some elongated interstitial cells in bronchial areas, consistent with fibroblasts, and in some isolated bronchiolar epithelial cells (not shown). In comparison, in JSRV-infected lung slices SU-positive cells aligned with P-ERK1/2 labeling cells in 18 of 41 of the SU-positive “nodules” observed (Figure 7F, I). In uninfected and JSRV-ΔRT-infected lung slices the majority of sections were completely negative for P-ERK1/2 (Figure 7L). Rarely, some interstitial cells were positive for P-ERK1/2 in both cytoplasmic and nuclear compartments (not shown), and in one lung slice a few bronchiolar ciliated epithelial cells were labeled but there was no labeling of alveolar epithelial cells (not shown). Taken together, these data indicate that JSRV-infected cells in lung slices exhibit increased proliferation and activation of Akt and ERK1/2, which closely resembles the phenotype of OPA tumor cells *in vivo*.

### Effect of age of donor animal on JSRV infection of lung slices

**Figure 8:**
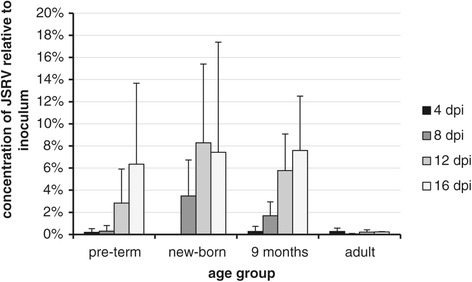
**Replication of JSRV is more efficient in lung slices from younger sheep.** JSRV was added to lung slices obtained from donor animals of different ages and the medium changed daily. The results show JSRV RNA content of 24 h supernatants estimated by RT-qPCR and presented as a percentage of the inoculum. The results represent data from pools of 4 lung slices in each of 3 replicate experiments for each age group. JSRV-∆RT-infected controls are not shown as these were all negative after d 4. Control lung slices without virus were negative for JSRV RNA throughout. The pre-term, new-born and 9 months groups show increasing values with greater dpi, whilst the adult group remains low throughout.

The large variability in efficiency of JSRV infection in lung slices led us to ask whether the developmental stage of the lung could influence susceptibility to JSRV infection in this *ex vivo* culture system, as an age-dependent effect in the development of OPA in experimentally infected sheep has been demonstrated previously [35]. Lung slices were prepared from 3 animals of each of 4 age groups: pre-term (1 week prior to expected parturition), new-born (1–5 days old) and nine month old lambs, and adults (2–10 years) and infected with JSRV or JSRV-∆RT. RT-qPCR analysis of supernatants of JSRV-infected lung slices suggested that those from adult sheep produced less JSRV than lung slices from the younger age groups (Figure 8). Statistically significant differences were found between the adult group and the new-born (p = 0.0253) and 9 months (p < 0.0001) groups. The 9 months group was also statistically significantly different from the other groups; pre-term p = 0.0014, new-born p = 0.0155.

## Discussion

In this study we have demonstrated the *ex vivo* infection of cultured ovine lung tissue with JSRV and the development of small proliferating foci of cells with properties similar to OPA lesions *in vivo*. Several features of this system replicate the properties of natural and experimental infections of lambs with JSRV, including the phenotype of the target cell and the activation of Akt and ERK1/2 signaling pathways (IHC data is summarized in Table 2). The similarity of cells in JSRV-infected foci in lung slices with those found in OPA *in vivo* suggests that they may represent early tumors. However, further studies are necessary to establish the tumorigenic nature of the cells; for example, by formation of tumors after transplantation into athymic nude mice as was demonstrated for OPA cells derived from clinical cases [36,37]. Nevertheless, we propose that lung slice infection represents an authentic *in vitro* model for studying the early events of JSRV infection and transformation.

**Table 2:** **Summary of IHC labeling of JSRV-infected cells in lung tissue samples from natural cases of OPA** (**OPA-N) compared with JSRV infected and uninfected ovine lung or lung slices**

**Antibody target antigen**	**Uninfected lung or lung slices**	**Tumor cells (OPA-N)**	**JSRV-positive lung slice cells**
Cytokeratin	Epithelial cells	Positive	Positive
JSRV SU	Negative	Positive	Positive
Club cell specific protein (CCSP)	Club cells in bronchial and bronchiolar epithelium	Negative	Negative
DC-LAMP	Type II pneumocytes	Mostly positive Mild to intense	Mostly positive Mild to intense
Ki-67	Very few cells in lung except macrophages. In lung slice, cells around edges	A few positive cells	Many positive cells
Phospho-Akt (Ser473)	Bronchiolar epithelial cells and some macrophages	50-100% positive Mild to intense	6/6 lung slices positive intense
Phospho-ERK1/2 (Thr202/Tyr204)	Endothelial cells, some fibroblasts, some bronchiolar cells	All samples positive. Individual OPA nodules varied from mild to intense	3/6 lung slices positive mild
Normal rabbit serum	Negative	Negative	Negative
Mouse IgG	Negative	Negative	Negative

### Previous *in vitro* systems for studying JSRV

Previous *in vitro* studies on JSRV have been limited by the lack of a permissive cell line that can support efficient virus replication. One reason for this is that the majority of cell lines tested do not support transcription from the JSRV LTR, which requires transcription factors that are preferentially expressed in type II alveolar pneumocytes and bronchiolar club cells [38]. Attempts to infect ovine cell lines with JSRV have achieved low levels of replication and even in those cells JSRV could only be detected by PCR for proviral DNA or by ultrasensitive assays for reverse transcriptase activity, and only after several passages of cells [39]. A further difficulty is that primary type II pneumocytes and club cells do not maintain their differentiated phenotype when cultured as monolayers on a plastic substrate [40]. This altered differentiation state in monolayer culture is mirrored by OPA tumor cells, which stop producing JSRV after a relatively low number of passages *in vitro* [41]. Interestingly, the expression of JSRV and of surfactant proteins can be reactivated by growing OPA tumor cells in a 3D culture system as spheres of polarized cells [42] and the differentiated phenotype of primary ovine lung cells can be prolonged for several passages in culture by growth on a 3D matrix [25]. These findings demonstrate that polarization of epithelial cells is important for maintaining their differentiated phenotype *in vitro.* Because JSRV replication depends on this differentiated state, 2D cultures are of limited usefulness for studying JSRV infection. Additionally, it is well established that tissue architecture and interactions with other cell types have a significant effect on the way cells respond to infection or external signals (reviewed by [43]). Such responses may include intracellular signaling pathways activated by JSRV Env and in this respect it is notable that previous studies have shown that the signaling pathways activated by JSRV in cell lines differ depending on the cell line used [13,44] and on whether the cells are grown in monolayer or 3D culture. By providing a source of stably differentiated type II pneumocytes, together with other cell types in the lung parenchyma, the lung slice culture system described here provides an ideal platform for studying the interaction of JSRV and its host cell *in vitro* and is likely to more accurately represent the pathways activated in natural tumors than studies in cell monolayers. Furthermore, the lung slice system allows very early events in infection to be studied *in vitro*. Such studies are challenging *in vivo* due to difficulty of finding small infected foci within the large volume of the lung. Moreover, the lung slice system permits many replicate slices to be prepared from a single animal, thereby reducing the number of animals required compared to *in vivo* studies.

### Replication of JSRV

Evidence that JSRV is able to infect and replicate in sheep lung slice cultures was provided by the detection of JSRV RNA in the supernatants of lung slices (Figure 2) and by the appearance of JSRV SU-positive cells detected by IHC. In most infected slices, JSRV production was maintained and increased up to 16 or 20 days in culture at which point the experiments were terminated. The increase in the number of SU-positive groups of cells over time, in addition to expansion of the number of positive cells per group, suggests that additional rounds of infection occurred in the lung slices secondary to the primary inoculum. This is supported by the observation that JSRV released by infected lung slices is infectious (Figure 3). However, we cannot rule out that cells infected from the inoculum may have become SU-positive at different rates, nor that infected cells may have migrated to form new foci within the lung slices.

Although this system clearly provides a valuable platform for studying JSRV, it was notable that virus production in lung slices was highly variable, both between different donor animals and between replicate slices from the same lung. The low amount of viral RNA in some lung slice supernatants approached the limit of sensitivity of the RT-qPCR, which likely contributed to the variability of the amount of virus detected (Figure 2) [45]. However, the variation between replicate lung slices was also evident from IHC labeling which, although not quantitative, showed that some lung slices had many SU-positive cells whereas others from the same donor lung infected at the same time with the same JSRV virus stock had few or none. There is, of course, natural variability in the precise numbers of different cell types between each individual lung slice; for example, the proportion of alveoli compared to conducting airways is different in each slice and this may, to some degree, explain the variability of infection observed. Future studies will examine the variable infectivity of JSRV in lung slices further, with the aim of increasing the reproducibility of the system.

### Identity of cell types infected with JSRV

An important factor determining JSRV infection in lung tissue is the availability of appropriate target cells. The identity of cell types infected and transformed by JSRV is of great interest as they may be relevant to parallel studies in mice and humans focused on identification of the cells of origin in lung cancer [46]. Here, we focused only on those cells that expressed JSRV SU protein detectable by IHC. We recognize that JSRV may infect some cells in lung tissue non-productively, for example where the virus enters and integrates but where expression of viral proteins does not occur. It is conceivable that such cells may act as a latent reservoir of infection in infected sheep but for the purposes of this study we regard them as irrelevant to pathogenesis.

Early studies on natural OPA indicated that the tumor derives from type II pneumocytes in alveoli and, less commonly, from bronchiolar club cells. This conclusion was based on ultrastructural studies showing viral particles in cells [47,48] and immunohistochemical analysis that suggested the majority of OPA tumor cells express SP-C and a minority express CCSP [30].

More recent work on natural and experimentally-derived OPA tumors has used multi-colored immunofluorescence and IHC to identify the phenotype of JSRV-positive cells more definitively. These studies show clearly that the large majority of JSRV-positive cells within OPA tumors co-express SP-C, indicating that they are cells of the type II pneumocyte lineage [18,19,22]. However, some of these studies also identified CCSP and JSRV Env double-positive cells either in natural tumors [22] or as solitary cells in experimentally-infected lambs 10 dpi [18], indicating that ovine club cells can also be infected by JSRV and express the viral oncoprotein. These data confirm that transformation occurs primarily in SPC-positive cells in the type II pneumocyte lineage, whereas JSRV infection of club cells may contribute a relatively minor part to tumor growth.

Here, we investigated which cell types are infected by JSRV in cultured lung slices and how this compares with *in vivo* infection. Using IHC labeling of serial sections, we found that groups of cells expressing JSRV also expressed markers of type II pneumocytes. Similar labeling was found in OPA-N where OPA tumor cells labeled positively for JSRV SU, cytokeratin, and DC- LAMP. In contrast, we did not find OPA-N tumor cells that were positive for CCSP. Similarly, JSRV-infected cells in cultured lung slices also showed no evidence for expression of CCSP. The localization of JSRV infection to the same cell type in tumors and cultured lung slices further strengthens the utility of this *ex vivo* system as a model for OPA.

In common with most other retroviruses, JSRV is able to infect dividing cells much more efficiently than non-dividing cells [19,49]. Therefore, the abundance of dividing type II pneumocytes is likely to be a major determinant of the ability of the virus to infect and replicate in lung tissue, both *in vivo* and *in vitro*. Previous work using experimentally-infected lambs has demonstrated that younger animals are more susceptible to infection and disease progression and have a shorter incubation period than older animals [35]. As no significant adaptive immune response is raised against JSRV in infected sheep, it was hypothesized that the different susceptibility *in vivo* may instead be due to the availability of target cells which may be age-dependent. There is an increase in the proportion of type II pneumocytes during the large increase in lung volume and gas exchange surface area in the first weeks of life [50,51] and although adult sheep appear refractory to JSRV infection and tumor development, they become susceptible following experimental lung injury, which induces tissue repair and increased cell division of type II pneumocytes [19]. It has been suggested [19,21] that in naturally infected sheep, lung repair in response to injury, for example, by bacterial infection, may be important to allow JSRV to move from cells of the lymphoreticular system where replication is very low into the target cells in lung for JSRV transformation and replication.

Our data from lung slice infections show a similar trend to *in vivo* studies, in that lung slices established from adult sheep appeared to be less susceptible to JSRV infection *in vitro* than slices taken from lambs (Figure 8). This is most likely to be a reflection of the abundance of suitable target cells, (dividing type II pneumocytes), but we cannot rule out that there may also be additional age-related host differences that influence the ability of JSRV to infect and replicate such as the expression of anti-viral restriction factors or mediators of innate immunity. Further studies are needed to examine this question quantitatively. Nevertheless, the available data indicate that to have the best chance of establishing JSRV infection in lung slices, the use of lung tissue from younger sheep is preferable.

### Transformation by JSRV

Previous studies have demonstrated that JSRV oncogenesis is mediated by the viral Env protein which activates protein kinase signaling pathways involved in transformation [13]. In the present study, we found that tumor cells in OPA-N and groups of cells expressing JSRV in infected lung slices had a higher proportion of proliferating cells than uninfected lung or lung slices, as determined by Ki-67 labeling (Figure 6). This suggests that JSRV Env expression in lung slices can stimulate proliferation as it does in naturally infected lung [17] and indicates that the lung slice model can be used to study mechanisms of JSRV transformation *in vitro*.

The precise mechanism by which JSRV Env drives transformation is unclear, but it is thought to activate a number of cell signaling pathways that control cell growth and differentiation. As noted above, the specific pathways activated depend on the model used [13]. To have value as an *in vitro* model, JSRV should activate the same signaling pathways in cultured lung slices as it does in natural tumors. Here, we examined activation of Akt and ERK1/2 (MAPK p42/p44) as these pathways have been identified in most previous studies of transformation with JSRV and are also important in human lung adenocarcinoma [13,52,53].

Dysregulation of Akt signaling pathways is common to many cancers [54,55]. Phosphorylation (activation) of Akt allows it to dissociate from the plasma membrane and move to the cytoplasm or nucleus where it can activate downstream pathways involved in cell growth, division, survival, and metastasis [33,34]. Our IHC analysis indicated that JSRV-infected cells in OPA-N and in lung slices labeled positively for P-Akt (Figure 7). Therefore, *in vivo* and *in vitro*, JSRV infection correlates with the presence of P-Akt. This finding is in agreement with studies on isolated OPA tumor cells grown in 3D culture where Akt signaling was reported to be dysregulated [56]. However, our finding that OPA tumors are positive for P-Akt is in contrast to a previous study which reported that OPA tumors do not express activated Akt [57]. The reason for this apparent discrepancy is unclear as the same commercial antibody was used here as in that study.

ERK1/2 activation has been described in natural and experimentally derived OPA tumor tissue [44,58] and in OPA-derived tumor cell lines (Maeda and Fan unpublished, cited in [44]). Our data from JSRV-infected lung slices suggest that activated ERK1/2 is present in some JSRV-infected cells but that it is found less consistently than P-Akt. It is possible that activation of the ERK1/2 pathway may occur later in transformation than Akt activation. Alternatively, the detection of P-ERK1/2 may be dependent on whether inhibitory regulatory pathways, such as MAPK p38, are also active, as these have also been reported to be activated by JSRV Env in cell line studies [44]. While further analysis is necessary, it appears likely that additional pathways may be active in established tumors compared to “new” infections or that factors present *in vivo* but not *in vitro* result in increased P-ERK1/2. Such factors include the presence of infiltrating myeloid cells, which are a key feature of OPA-N. In many other cancers, tumor-associated macrophages and neutrophils have important roles both in modulating tumor cell growth and in potentiating the local anti-tumor immune response [59]. While infiltrating cells are not present in lung slices as these are derived from healthy sheep, it would be interesting to collect these, by broncho-alveolar lavage, from OPA positive sheep and introduce them during the culture period to examine their effect on infection and transformation. Thus the lung slice model described here provides an ideal platform for future studies to dissect the temporal changes in pathway signaling and to examine other aspects of JSRV infection and transformation.

## Conclusions

We have shown that JSRV infects type II pneumocytes in ovine lung slice cultures resulting in the production of infectious virus particles and the activation of signaling pathways associated with cell proliferation. Collectively, these data demonstrate that JSRV infection of lung slices cultured *ex vivo* reproduces many of the features of JSRV infection of the ovine lung *in vivo*. This model therefore provides a tractable *in vitro* system for studies on JSRV replication and pathogenesis that will permit detailed dissection of the early molecular events occurring in JSRV-induced oncogenesis. Furthermore, the availability of the lung slice system will further strengthen the utility of OPA as a model for human lung adenocarcinoma. Finally, we note that the lung slice system could be utilized for studying the effects of other oncogenes known to be important in lung cancer such as mutated *KRAS* and *EGFR* [53,60] and may also find application in the *in vitro* evaluation of cancer therapeutics.

## Methods

### Animals

All animal work was performed at the Moredun Research Institute in accordance with local ethics committee approval and UK Home Office regulations. Tissue slices of normal ovine lung were prepared from lungs obtained from healthy sheep that were either untreated negative control animals in other experiments or animals culled as part of the normal management of our high health status flock. Tissue slices of OPA lung tumor were prepared also from natural cases of OPA (OPA-N) that were donated by farmers and brought to the Moredun Research Institute for post-mortem examination and definitive diagnosis. Experimentally-induced cases of OPA (OPA-E), used for comparison by identical immunohistochemistry (see below), were from archived formalin fixed paraffin-wax embedded lung samples from previous studies [18].

### Preparation, culture and JSRV infection of ovine lung slices

Animals were killed by captive bolt followed by immediate exsanguination. Lungs were removed whole along with the trachea and heart immediately after death. Using a 50 ml syringe, low melting point agarose (Sigma type IX-A, 2% in Hank’s Balanced Salt Solution (HBSS; 5.4 mM KCl, 0.44 mM KH_2_PO_4_, 4 mM NaHCO_3_, 0.14 M NaCl, 3.4 mM NaH_2_PO_4_, 5.6 mM D-Glucose)), at 37°C was introduced via a mainstem bronchus to a whole lung (fetuses and lambs) or a single lung lobe (adult animals; right cranial or right middle), until moderate expansion was achieved. The agarose-infused lung/lobe was clamped off at the primary bronchus, dissected away from the rest of the lung and placed immediately into HBSS on ice for 1 hour. A Krumdieck tissue slicer (Alabama Research and Development, Munford, AL) was used to cut slices of lung parenchyma approximately 300 μm thick and 8 mm in diameter. These were washed 5 times with lung slice wash medium (LSWM; DMEM, 50 U/ml penicillin/streptomycin, 4 mM L-glutamine, 0.2 μg/ml Gentamicin, 1.25 μg/ml Amphotericin B (all Sigma)) and transferred into individual wells of a 24-well tissue culture plate containing 0.3 ml per well of epithelial cell medium (ECM; Quantum 286 (PAA Laboratories Ltd), 50 U/ml penicillin/streptomycin, 4 mM L-glutamine, 0.2 μg/ml gentamicin, 1.25 μg/ml amphotericin, 5 ng/ml recombinant human hepatocyte growth factor, 10 ng/ml recombinant human keratinocyte growth factor, 10 μM 8-bromo-adenosine 3′,5′-cyclic monophosphate, 100 μM 3-isobutyl-1-methylxanthine, 100 nM dexamethasone (all Sigma)). Control wells containing no lung slice (NLS) were also included on each plate. Lung tumor slices were prepared as for lung tissue except that the OPA-affected tissue was sufficiently dense that it did not require addition of agarose prior to cutting.

The plates were incubated in a humidified incubator at 39°C, 5% CO_2_ on an inclined rotator (Alabama Research and Development, Munford, AL) set at 1 revolution per minute (rpm). After 24 hours, the lung slices and NLS wells were washed three times with 0.5 ml LSWM per well. To each well was added 150 μL of ECM containing 8 μg/ml polybrene (Sigma) and approximately 6 × 10^6^ RNA copies per ml of JSRV (JSRV or JSRV-∆RT, see below) and incubated at 39°C, 5% CO_2_, 1 rpm for 2 hours when an additional 150 μl of ECM was added. As there is no permissive cell line available for JSRV, infectious titers cannot be determined, therefore the RNA copy number of JSRV and JSRV-∆RT in the inocula was estimated as described previously [61]. Virus was removed after a total of 24 hours and the wells were washed 3 times with LSWM before adding 300 μl of ECM. Medium was harvested and replaced every 24–36 hours thereafter. A further control of 4 lung slices without JSRV challenge was included in each 24-well plate.

At 4 day intervals four lung slices each from the uninfected, JSRV-infected or JSRV-∆RT-treated groups were collected and fixed in 10% buffered neutral formalin for subsequent immunohistochemistry (IHC). Also, at each time point lung slice supernatant (*i.e.*, the spent medium) from 24 hours of culture was retained; supernatants were pooled from 4 wells and centrifuged at 12,000 ×g for 2 minutes to remove cells and cell debris before aliquoting and storing at −80°C.

For three lung slice experiments, supernatants from the JSRV-infected or JSRV-∆RT-treated lung slices were collected daily from 11 to 16 dpi and were prepared as above and stored at −80°C. These were thawed, pooled and concentrated by centrifugation at 50,000 × g for 2 hours and the pellet was resuspended in 1/40 volume of ECM. In duplicate experiments these concentrated supernatants were used to infect lung slices essentially as described above except that 50 μl was used as inoculum per well (viral RNA copy number was not determined).

### Virus production

Infectious wild-type JSRV was produced by *in vitro* transfection of 293 T cells with plasmid pCMV2JS_21_ as described [18]. Culture supernatants were harvested 48 and 72 hours post-transfection, filtered (0.45 μm), aliquoted and stored at −80°C for use in the entire series of experiments. As a negative control virus, we generated a replication-defective molecular clone of JSRV that contains a mutation in the active site of reverse transcriptase (YMDD to AAGA). This mutant (JSRV-∆RT) was generated by PCR of pCMV2JS_21_ using 5′-phosphorylated primers 5′-GCCGCTATATTACTAGCTCATGCTG-3′ and 5-GCCGGCATGAACCAAGTATAGCTGAGG-3′ with the KOD High Fidelity polymerase system (Merck) as recommended by the manufacturers. The resulting 11.5 kb product was re-circularized by ligation and used to transform *E. coli* XL10-Gold cells (Stratagene). DNA sequencing of the resulting clones was performed to confirm the desired mutation and the absence of polymerase errors. JSRV-∆RT virions were prepared in the same way as wild type JSRV.

### Cell viability analysis of lung slices

Visualization of active coordinated movement of cilia on the ciliated respiratory epithelial cells was used as an indicator of the viability of the lung slices [27]. In addition, viability of cells was evaluated on some of the lung slices by treatment with LIVE/DEAD reduced biohazard viability/cytotoxicity assay reagents (Molecular Probes, 768736). The stock reagents (SYTO 10 green fluorescent nucleic acid stain and DEAD Red ethidium homodimer-2 nucleic acid stain) were diluted to their final working concentrations (1:500) in HBSS. Each slice was incubated in 200 μl of solution at room temperature for 30 minutes. As a positive control for the dead stain, one set of lung slices was treated with 1% Triton X-100 for 15 minutes prior to staining and subsequent imaging.

### Reverse transcriptase-quantitative PCR (RT-qPCR)

RT-qPCR was performed as described previously [61]. Briefly, RNA was extracted from 140 μl of lung slice supernatant (pooled from 4 wells which each contained a lung slice) using the QIAmp viral RNA mini kit (QIAgen) exactly as manufacturer’s instructions and eluted in 50 μl of the buffer provided. Contaminating DNA was removed using the TURBO DNA-free kit (Ambion) prior to RT-qPCR by adding 1.5 μl 10× DNase buffer, 1 μl water and 0.5 μl TURBO DNase to 12 μl of RNA and incubating at 37°C for 30 minutes. DNAse inactivation reagent (1 μl; Ambion) was added, vortexed several times and then centrifuged. 5 μl of the supernatant was used in duplicate RT-qPCR reactions. LTR primers, fluorescent labeled probe, and RT-qPCR conditions were as described previously [61].

Stored aliquots of day 4 supernatant from a lung tumor slice culture were used as an extraction positive control. An RNA standard curve was generated as described previously [61]. The concentration of virus in the supernatants was expressed relative to the concentration of virus in the inoculum as E^∆Ct^ where E is the amplification efficiency calculated from the slope of the standard curve according to the formula: E = 10^(−1/slope)^ and ∆Ct is the difference between the Ct values for the d0 and dn supernatants.

### Statistical analysis

To statistically analyze the differences between lung slices from animals of different ages a linear model was fitted using generalized least squares (GLS) by REML allowing heterogeneous group variances. The model was applied on the rank-based inverse normal transformation of the data where age group and dpi were, respectively, main effect and co-variate. For pairwise comparison multiple group comparisons were arranged from the fitted model and the resulting p-values were adjusted to control for the false discovery rate.

### Immunohistochemistry

Lung tissue and lung slices were fixed in 10% neutral buffered formalin, processed routinely by dehydrating through graded alcohols, embedded in paraffin wax, sectioned (5 μm) and mounted on Superfrost™ glass microscope slides (Menzel-Gläser, Braunschweig, Germany). IHC was carried out as described previously [18]. The optimal concentration of each primary antibody was ascertained using appropriate positive control tissue as listed in Table 1. Negative controls for IHC were performed by substitution of the primary antibody with purified mouse IgG, rabbit IgG, or normal rabbit serum as appropriate. IHC was performed on sections of lung that contained areas of OPA identified by standard histological examination (hematoxylin and eosin (HE) staining). The sections were from six sheep that were naturally affected and had advanced clinical signs of OPA and six that had been experimentally infected with JSRV and had early stage asymptomatic OPA, where tumors were visible histologically but were not sufficiently advanced to induce clinical signs. IHC was performed on sections of OPA-N and OPA-E lung tissue, lung slices and lung tumor slices. For IHC of consecutive serial sections with different antibodies (Table 1) JSRV-infected lung slices with more than 3 groups of SU-positive cells were selected; these lung slices had been prepared from 6 different animals of different ages (5d to 10 years) and were harvested for IHC at 16 or 20 dpi. The equivalent JSRV-∆RT-treated lung slices were used as negative controls.

## Abbreviations

dpi: Days post infection
IHC: Immunohistochemistry
JSRV: Jaagsiekte sheep retrovirus
OPA: Ovine pulmonary adenocarcinoma
OPA-E: Experimentally-induced OPA
OPA-N: Natural OPA
P-Akt: Phosphorylated Akt
P-ERK1/2: Phosphorylated extracellular-signal-regulated kinase 1/2
RT-qPCR: Quantitative reverse transcriptase polymerase chain reaction
